# 530. Population Pharmacokinetic Analysis of Ensitrelvir, an Inhibitor of 3C-like (3CL) Protease of Severe Acute Respiratory Syndrome Coronavirus 2 (SARS-CoV-2) for patients with SARS-Cov-2 Infection

**DOI:** 10.1093/ofid/ofad500.599

**Published:** 2023-11-27

**Authors:** Toru Ishibashi, Luna Nomura, Ryosuke Shimizu, Ryuji Kubota

**Affiliations:** Shionogi & Co., Ltd., Osaka, Osaka, Japan; SHIONOGI & CO., LTD., Osaka, Osaka, Japan; Clinical Pharmacology & Pharmacokinetics, Project Management Department, Shionogi & Co., Ltd. Osaka, Japan, Osaka, Osaka, Japan; Clinical Pharmacology & Pharmacokinetics, Project Management Department, Shionogi & Co., Ltd. Osaka, Japan, Osaka, Osaka, Japan

## Abstract

**Background:**

Ensitrelvir is a novel oral inhibitor of 3CL protease of SARS-CoV-2, which is essential for viral replication. Ensitrelvir which is administered at once daily for 5 days with 375 mg on Day 1 followed by 125 mg, was approved in Japan for the treatment SARS-CoV-2 infection under the Emergency Regulatory Approval System in November 2022. The aim of this study is to build a population pharmacokinetic (PK) model to assess covariates on ensitrelvir PK.

**Methods:**

A population PK analysis was performed using a total of 8034 plasma ensitrelvir concentrations from 2060 healthy participants in Phase 1 studies and patients in Phase 2/3 study. A 2-compartment model with first order absorption was used as a structural PK model for a base model. The effects of covariates were evaluated including age, body weight (BWT), AST, ALT, albumin, total bilirubin, creatinine clearance, GFR, sex, race, health status, formulation, food conditions and so on. The exposures (individual post-hoc maximum concentration [Cmax], area under the time-concentration [AUC] and plasma concentration at 24 hours post-dose [C24]) in the patients were estimated with an empirical Bayesian approach based on the final model to assess the pharmacokinetic/pharmacodynamic (PK/PD) relationship between SARS-CoV-2 viral RNA load and exposures.

**Results:**

The population PK model well described the plasma ensitrelvir concentration data. The effects of BWT on clearance and volume of distribution, food conditions and formulation on absorption rate constant were found to be significant covariates. The anti-viral effect did not change in the range of ensitrelvir exposure in Phase 2/3 study, while the efficacy of ensitrelvir was demonstrated by significant reduction of viral RNA compared with placebo.

**Conclusion:**

The population PK model was developed based on the plasma ensitrelvir concentration data from participants including patients infected with SARS-CoV-2 and the BWT was the most important covariate on ensitrelvir PK. The expected exposures for anti-viral effect at the clinical dose were obtained regardless BWT. The population PK model would be useful for understanding PK characteristics of ensitrelvir and the PK/PD results supported that the clinical dose of 375/125 mg is appropriate for treatment of SARS-CoV-2 infection.

**Disclosures:**

**All Authors**: No reported disclosures

